# Extending the Bicriterion Approach for Anticlustering: Exact and Hybrid Approaches

**DOI:** 10.1017/psy.2025.10052

**Published:** 2025-10-07

**Authors:** Martin Papenberg, Martin Breuer, Max Diekhoff, Nguyen K Tran, Gunnar W Klau

**Affiliations:** 1 Department of Experimental Psychology, Heinrich Heine University Düsseldorf, Germany; 2 Department of Computer Science, Heinrich Heine University Düsseldorf, Germany; 3 Centre for Digital Medicine, Heinrich Heine University Düsseldorf, Germany

**Keywords:** anticlustering, bicriterion optimization, exact algorithms, maximum dispersion

## Abstract

Numerous applications in psychological research require that a data set is partitioned via the inverse of a clustering criterion. This anticlustering seeks for high similarity between groups (maximum diversity) or high pairwise dissimilarity within groups (maximum dispersion). Brusco et al. (2020) proposed a bicriterion heuristic (BILS) that simultaneously seeks for maximum diversity and dispersion, introducing the bicriterion approach for anticlustering. We investigate if the bicriterion approach can be improved using exact algorithms that guarantee globally optimal criterion values. Despite the theoretical computational intractability of anticlustering, we present a new exact algorithm for maximum dispersion that scales to quite large data sets ( 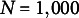 ). However, a fully exact bicriterion approach was only feasible for small data sets (about 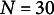 ). We therefore developed hybrid approaches that maintain optimal dispersion but use heuristics to maximize diversity on top of it. In a simulation study and an example application, we compared several hybrid approaches. An adaptation of BILS that initiates each iteration with a partition having optimal dispersion (BILS-Hybrid-All) performed best across a variety of data inputs. All of the methods developed here as well as the original BILS algorithm are available via the free and open-source R package anticlust.

## 1 Introduction

Partitioning data sets via anticlustering establishes homogeneity between clusters and heterogeneity within clusters. Applications exist in many research fields, including psychology (Brusco et al., 2020; Nagel et al., 2024; Schaper et al., 2023), test assembly (Gierl et al., 2017), education (Baker & Powell, 2002; Krauss et al., 2013), artificial intelligence (Steghöfer et al., 2013), machine learning (Mauri et al., 2023; Rahu et al., 2024), network systems (Mohebi et al., 2022), and operations research (Gallego et al., 2013; Gliesch & Ritt, 2021). In psychological research, an important application is the assignment of stimuli to different experimental sets that are presented in within-subjects designs: The different sets should be as similar as possible with regard to variables that affect the responses (Lahl & Pietrowsky, 2006). Anticlustering is a powerful tool for automatically assigning stimuli—or in general, any objects—to equivalent subsets (Papenberg & Klau, 2021). Handling this task manually, on the other hand, is time-consuming and usually inadequate (Cutler, 1981; Lintz et al., 2021).

In the current article, we investigate and extend a bicriterion anticlustering method that was recently introduced by Brusco et al. (2020). Their algorithm aims for group-level similarity between clusters (via the diversity criterion) and heterogeneity between pairs of objects in the same cluster (via the dispersion criterion). We investigate how their bicriterion approach profits from employing exact algorithms as opposed to the pure heuristic approach that was originally presented by the authors. Our article is organized as follows. We start by formalizing the computation of anticlustering objectives, before reviewing exact and heuristic algorithms for optimizing these objectives. We reiterate the bicriterion heuristic by Brusco et al. (2020) and then present our own contributions to the bicriterion approach. We evaluate our extensions using synthetic data in two simulation studies as well as real data in an example application. All anticlustering methods presented in this article are available as part of the free and open-source R package anticlust (Papenberg & Klau, 2021). All code and data used in the simulations and practical examples are available from the accompanying Open Science Framework (OSF) repository (https://osf.io/hsztn/).

### 1.1 Formalizing anticlustering

Anticlustering is used to partition *N* objects into *K* exhaustive and mutually exclusive subgroups. Following the notation by Brusco & Cradit (2005), we define 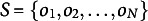 as a set of *N* objects and 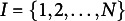 as the set of their indices. A partition of a set *S* into *K* clusters is denoted as 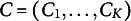 , where the elements of cluster 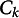 are the indices of objects assigned to cluster *k* and the number of objects assigned to cluster *k* is 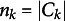 ( 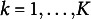 ). A feasible partition of the *N* objects is defined via (a) 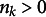 for all 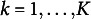 , (b) 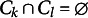 for 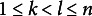 , and (c) 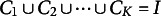 . We denote the set of feasible partitions of *N* objects into *K* clusters as 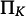 ( 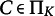 ). In anticlustering, *K* and 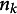 are usually predetermined by the application. We will refer to the subgroups 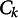 as anticlusters, clusters, or groups interchangeably, and to the collection *C* as the anticlustering partitioning. We will refer to the fixed values 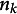 as cardinality constraints.

Anticlustering seeks for homogeneity between groups and heterogeneity within groups by maximizing a clustering objective criterion. Thus, anticlustering is the logical and mathematical opposite of classical clustering, which partitions data sets via minimization of the same criteria (Brusco et al., 2020; Späth, 1986; Steinley, 2006). We assume that a data matrix 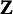 is used as input for computing the anticlustering criterion on the basis of a partitioning *C*. 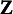 is either a feature matrix 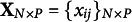 , where the *P* columns contain numeric or categorical measurements on the *N* objects, or a symmetric matrix 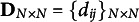 of pairwise dissimilarity measurements between the objects. The partitioning is then obtained by maximizing a clustering criterion 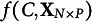 or 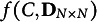 .

#### 1.1.1 The k-means criterion

The *k*-means criterion is computed as the sum of squared Euclidean distances between each object and its cluster center, also coined the *variance* (Späth, 1986; Steinley, 2006): 
(1)

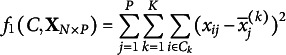



(2)

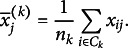



The *k*th cluster center 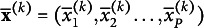 contains the mean values of the *P* variables computed across all observations belonging to the *k*th group. There is a direct connection between 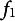 and the location of the cluster centroids (Späth, 1986). When minimizing 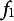 —the goal of *k*-means cluster analysis—the centroids 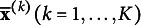 are as far located as possible from the global centroid 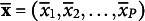 ; 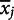 ( 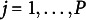 ) is computed as the global mean of the *j*’th attribute: 

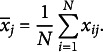



When maximizing 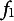 , which is the goal of *k*-means anticlustering, the cluster centroids are as close as possible to the global centroid 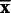 —and as a consequence to each other. In particular, maximizing 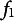 is equivalent to minimizing the weighted sum of the squared Euclidean distances between the global centroid and the cluster centroids (Steinley, 2006):^1^

(3)

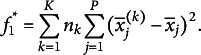



Thus, *k*-means anticlustering directly maximizes the similarity of the mean attribute values between clusters. Though counterintuitive, the connection between mean values and the group variance is actually well-known in the context of Analysis of Variance (ANOVA). In ANOVA, the within-group variance is compared to the between-group variance to perform a statistical test among group means. If the variance within-groups is large relative to the variance between-groups, the group means are similar to each other. If the variance within-groups is small relative to the variance between-groups, the group means are dissimilar to each other, potentially leading to the conclusion that at least two group means are significantly different from each other. The *k*-means criterion is a multivariate extension of the ANOVA error sum of squares, as it can be computed on the basis up to *P* variables and not just one.

#### 1.1.2 The k-plus criterion

The *k*-plus criterion is an extension of the *k*-means criterion that can be implemented by adding fictitious variables to the data matrix 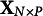 (Papenberg, 2024). When using the *k*-plus criterion, we do not only obtain similar means, but higher order moments, such as the variance or skewness as well. For example, to equate means and variances, we define a new data matrix 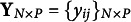 with 
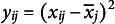
 , where 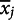 is the overall mean of the 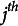 attribute. Then, the *k*-plus criterion is computed as 
(4)

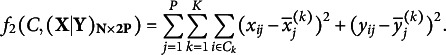



#### 1.1.3 The diversity criterion

Arguably, the most popular and important criterion for anticlustering is the *diversity*, which uses a dissimilarity matrix 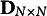 as input. It is computed as the within-group sum of pairwise dissimilarities across all clusters (Brusco et al., 2020; Feo & Khellaf, 1990; Gallego et al., 2013): 
(5)

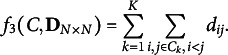

Maximizing the diversity 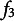 simultaneously minimizes the sum of distances between objects not in the same group. Therefore, the diversity represents within-group heterogeneity as well as between-group similarity (Feo & Khellaf, 1990). Being a sum instead of an average, however, it no longer adequately captures between-group similarity if group sizes are not equal (Papenberg, 2024).

#### 1.1.4 On the equivalence of variance and diversity: The average diversity

The diversity is most often computed by first converting a feature matrix 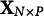 into a matrix of pairwise Euclidean distances 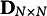 (Brusco et al., 2020; Gallego et al., 2013; Papenberg & Klau, 2021). If the squared Euclidean distances are used instead, we note an important equivalence between the *k*-means criterion 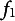 and the diversity 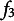 . Traditionally, the *k*-means criterion is denoted as the variance, i.e., the sum of squared Euclidean distances between data points and cluster centroids. However, it can also be expressed using the pairwise squared Euclidean distances among data points (Späth, 1980, p. 52). That is, “the sum of squared distances of a collection of points from the centroid associated with those points is equal to the sum of the pairwise squared distances between those points divided by the size (number of objects) of the collection” (Brusco, 2006, p. 350). Hence, if we let 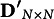 = { 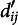 } represent the squared Euclidean distance, i.e., 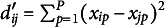 , the *k*-means criterion can be expressed as: 
(6)

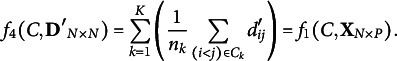

An important special case of anticlustering applications is that all groups are equal-sized, i.e., 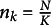 ( 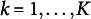 ). In this context, 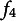 can be simplified to 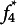 via 
(7)

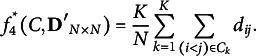

In the context of maximization, the factor 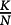 can be ignored because it does not depend on the partitioning *C*: 
(8)

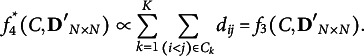

Therefore, the criterion 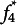 (i.e., the *k*-means criterion for equal-sized groups) reduces to the standard diversity 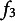 —if the diversity is computed on the basis of the squared Euclidean distance. For arbitrary measures of dissimilarity (and not just the squared Euclidean distance), we denote 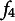 as the *average diversity* criterion, which was recently adapted by Mohebi et al. (2022).

#### 1.1.5 The dispersion criterion

Unlike 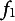 – 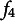 , the *dispersion* criterion does not quantify between-group similarity, but is a pure measure of within-group heterogeneity. It is defined as the minimum dissimilarity across any two objects that are part of the same group (Brusco et al., 2020; Fernández et al., 2013): 
(9)

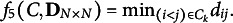

The dispersion is a measure of the “worst-case” pairwise dissimilarity (Brusco et al., 2020). Maximizing the dispersion ensures that any two objects in the same group are as dissimilar from each other as possible, which is desirable when striving for high within-group heterogeneity.

### 1.2 Algorithms for anticlustering

An anticlustering algorithm assigns objects to groups in such a way that one of the criteria 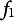 – 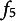 is maximized, while incorporating constraints on the number of groups and the number of objects per group (cardinality constraints). Anticlustering algorithms can be classified into *exact* methods on the one hand, and heuristics on the other hand. Exact algorithms are guaranteed to find a globally optimal partition according to the anticlustering criterion that is optimized. A globally optimal partition is a partition that has the highest value among all possible partitions. Heuristics do not make the promise of returning a globally optimal partition, but they usually provide satisfying results in practice (Papenberg & Klau, 2021), and their use is much more common. The preponderance of heuristics can be explained by the computational complexity of anticlustering. In the case of equal-sized groups, the number of possible anticlustering partitions 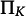 is computed as the normalized product of binomial coefficients (Papenberg & Klau, 2021): 
(10)

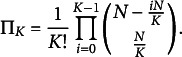




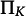
 grows exponentially with *N*, and even more severely so for higher values of *K*. For 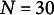 and 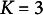 , there are already more than 900 billion possible partitions, which is beyond the search capacities of contemporary personal computers. Moreover, with the exception of some special cases, anticlustering problems have been identified as NP-hard problems (Feo & Khellaf, 1990; Fernández et al., 2013). A strong conjecture of theoretical computer science states that NP-hardness implies that with increasing problem size, “no amount of clever algorithm design can ever completely defeat the potential for combinatorial explosions that lurks within this problem” (Karp, 1986, p. 100). Thus, it is very likely that there is no algorithm that can find the globally optimal solution in running time that is a polynomial function of the input size, i.e., in “acceptable” running time (van Rooij, 2008). There are improved techniques, such as dynamic programming or integer linear programming (ILP) that may circumvent the computationally expensive explicit enumeration of all possible partitions (Brusco et al., 2024). These methods have the chance to return solutions for larger data sets than would be possible via complete enumeration. However, they do not prevent the eventual exponential explosion of the running time; instead, they just push the boundary of feasibility upward. The degree to which delaying the combinatorial explosion is successful depends on the specific problem or even the specific data input (Chen, 2017). For anticlustering, we even observe a sharp contrast in feasibility depending on the anticlustering objective: While partitions with optimal maximum dispersion can be found for quite large data sets (Fernández et al., 2013), creating partitions with optimal maximum diversity even fails for quite small data sets (Papenberg & Klau, 2021; Schulz, 2022).

#### 1.2.1 Exact algorithms for anticlustering

ILP is a powerful framework to optimally solve NP-hard combinatorial optimization problems. Brusco et al. (2025) recently provided an extensive overview of the usages of ILP in Psychology. Important applications are found in test assembly (Chen, 2017; van der Linden, 2005) or cluster analysis (Brusco et al., 2024). ILP uses mathematical modeling to present a problem as a combination of (a) decision variables (e.g., to encode if an object *i* is assigned to a cluster *k*), (b) an objective function to be optimized (e.g., the diversity), and (c) a system of constraints (e.g., to restrict the number of objects in a cluster). The model is usually formulated by a user and then given to a so-called solver, which returns values for the decision variables that yield the optimal value of the objective function. A variety of solvers exists, including commercial and open-source options. Commercial solvers, such as Gurobi (Gurobi Optimization LLC, 2024) oftentimes, outperform their open-source counterparts in terms of running time (Meindl & Templ, 2012).

Schulz (2022) contrasted three ILP models for finding anticlustering partitionings with maximum diversity: the standard formulation as described by Gallego et al. (2013), an adaptation of the clique partitioning model from Grötschel & Wakabayashi (1989) in Papenberg & Klau (2021), and a new block formulation. Schulz’s block formulation performed best and could solve problem instances up to 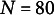 objects optimally within a time limit of 1,800 seconds. However, Schulz’s formulation is restricted to using the Manhattan distance as input, while, for example, the more common Euclidean distance cannot be used. The model by Papenberg & Klau (2021), which allows arbitrary measures of dissimilarity, was able to solve instances with up to 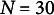 within the time limit, followed by the standard formulation, which solved problem sizes up to 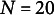 . These results are in contrast to the success of exact approaches in cluster analysis, where it is common that hundreds or even thousands of objects can be partitioned optimally in acceptable time (e.g., Böcker et al., 2011; Brusco, 2006; Brusco et al., 2024). Even though it is also NP-hard (at least when 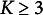 ; see Discussion), the maximum dispersion problem has been shown to be more accessible to exact solution approaches. Fernández et al. (2013) presented an exact algorithm based on ILP for a generalized version of the maximum dispersion problem that scaled to several hundred objects (see also Gliesch & Ritt, 2021).

#### 1.2.2 Heuristic algorithms for anticlustering

Heuristic methods for anticlustering broadly fall into the categories of local neighborhood search and metaheuristics. Applying the hill-climbing algorithm by Rubin (1967) to the domain of anticlustering, Späth (1986) used a single-object cluster relocation algorithm to maximize the *k*-means criterion. Starting with a random initial partition, each object is successively moved from its current cluster to all the other ones, and the difference in objective is computed for each of the exchanges. The one exchange that leads to the largest improvement in the criterion is actually realized. The exchange procedure is repeated for each object and, after iterating through all objects, is repeated from the beginning until no swap can improve the objective, i.e., until a local maximum is found. The entire process can be restarted multiple times based on different random partitionings. Because the hill-climbing method switches the cluster affiliation of individual objects, it yields a partitioning that aims at maximizing the *k*-means criterion over different possible group sizes—the group sizes are not known in advance. More commonly, however, anticlustering applications assume that group sizes are fixed. A straightforward adjustment of the hill-climbing method to accommodate fixed group sizes is to apply the exchange rule to pairs of objects, i.e., to inspect how exchanging two objects between their respective clusters influences the objective.^2^ This adaptation yields the method LCW by Weitz & Lakshminarayanan (1998), which at the time performed better than all of the available competitors. However, later research has focused on metaheuristics that tend to outperform simple local maximum search (e.g., Gallego et al., 2013). Different metaheuristics use different strategies for a more effective search through the available partitions, while trying to avoid getting stuck in local optima. Metaheuristics for anticlustering include variable neighborhood search (Urošević, 2014), tabu search (Gallego et al., 2013), iterated local search (ILS) (Brusco et al., 2020), simulated annealing, genetic algorithms (Palubeckis et al., 2011), or mixture approaches (Yang et al., 2022).

#### 1.2.3 The bicriterion heuristic for anticlustering

Brusco et al. (2020) outlined the importance of simultaneously considering multiple criteria when addressing anticlustering problems. In particular, they argued that anticlustering algorithms should simultaneously optimize an objective of between-group similarity, such as the diversity, and pairwise within-group dissimilarity, i.e., the dispersion. To this end, Brusco et al. (2020) presented a bicriterion algorithm (BILS) that simultaneously maximizes the diversity 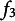 and the dispersion 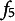 by approximating the Pareto efficient set of partitions according to both criteria. By investigating the Pareto set for many problem instances, they found that a considerable improvement in one of these criteria is often possible with little or even no sacrifice in the other.

The BILS is a two-phase algorithm. The initial phase performs a basic local maximum search (bicriterion pairwise interchange heuristic, BPI) similar to LCW, followed by a subsequent improvement phase via ILS, which attempts to escape local optima. The BPI guides its search via optimization of a weighted sum criterion of the diversity and dispersion, i.e., 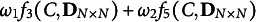 . By committing to exchanges that improve the weighted bicriterion, the BPI investigates the search space for partitions that have high diversity as well as high dispersion. The search is restarted repeatedly using a different initial random partitioning and varying 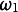 and 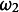 , yielding the multistart BPI or MBPI. Crucially, being a *direct* bicriterion algorithm (Ferligoj & Batagelj, 1992), the MBPI does not only conduct a search for improved bicriterion values, but an additional bookkeeping takes place during search: For each partition that is generated, a decision is made whether to include it in the Pareto set of efficient solutions. That is, the MBPI keeps a list of partitions that are not dominated by any other partition. A partition is dominated if there is another partition that is strictly better with regard to one criterion and not worse with regard to the other. After every exchange, the Pareto set is updated via potential inclusion of the new partition and potential removal of dominated partitions. The Pareto set is a set of partitions among which—without additional assumptions or quantification—no one partition is clearly better than any of the others; no Pareto partition is dominated by another Pareto partition with regard to both criteria (diversity and dispersion). The Pareto set is an important concept in multicriterion optimization and providing a Pareto set for dispersion and diversity was one of the main contributions of the original BILS algorithm.

The combination of the MBPI and the ILS yields the complete *bicriterion ILS* (BILS) algorithm. Building on a randomly selected partition from the Pareto set provided by the MBPI phase, the ILS iterates through all 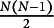 pairs of objects. For each pair, a decision is made whether to exchange their cluster affiliation. For each pair, the probability of a swap is uniformly sampled from the interval [5%, 10%]. This perturbation leads to a partition that is not locally optimal, which is then restored to local optimality using the BPI method. Because the perturbed partition tends to be similar to a “pretty good” locally optimal partition but is no longer bound by its local optimality, the BPI search has the chance of refining it toward an even better local optimum. As the BPI, the ILS can be restarted multiple times. Brusco et al. (2020) recommended using 10,000 restarts in total for the BILS (5,000 restarts of the BPI and 5,000 restarts of the ILS). The output of the BILS algorithm is the (approximated) Pareto set and not a single partition. From this Pareto set, users can select a partition according to their liking; Brusco et al. (2020) discussed several automated criteria for selecting a partition from the Pareto set, though the decision can also be based on substantive considerations.

### 1.3 Contributions of the current article

We endorse Brusco et al.’s bicriterion approach for anticlustering and extend it in several ways. First, in our software anticlust, we implemented several minor extensions that enhance BILS’s applicability. In our implementation, it is possible that the diversity and dispersion are computed on the basis of two different dissimilarity matrices 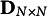 and 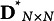 . Moreover, we allow optimizing the average diversity 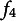 instead of only the ordinary diversity. The average diversity is better suited to represent between-cluster similarity when cluster sizes are unequal and via (6), allows the BILS to optimize the *k*-means and *k*-plus objectives. In the application presented below, we will make use of these extensions in the context of designing memory experiments.

As our most important contribution, we develop a model that yields provably optimal solutions for the bicriterion approach. Specifically, we employ a constraint method (Brusco et al., 2020; Brusco & Cradit, 2005; Brusco & Stahl, 2005) that first identifies an optimal value for maximum dispersion and then maximizes the diversity under the constraint of preserving maximum dispersion. The approach seamlessly generalizes toward generating the entire (optimal) Pareto set. After evaluating the exact bicriterion approach, we present hybrid approaches that aim for optimal maximum dispersion, but use heuristic algorithms to maximize the diversity.

### 1.4 OptDispF: An exact algorithm for maximum dispersion

To obtain a partitioning having maximum dispersion, we developed a new algorithm. The algorithm *opt*imally maximizes the *disp*ersion using *f*ixed group sizes, for which reason we refer to it as *OptDispF*. It is based on an approach employed by Brucker (1978) to solve the converse clustering problem for 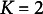 .

An important insight for finding an exact algorithm for maximum dispersion is that the dispersion acts like a set of cannot-link constraints on pairs of objects (Davidson, 2009). That is, if the pairwise dissimilarity of two objects is below some threshold 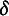 , they must not be linked in the same cluster. For the maximum dispersion problem, an exact algorithm has to find a 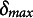 : the maximum possible 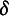 such that the resulting set of cannot-link constraints can still be satisfied, while for any value larger than 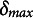 , at least one pair of objects has to be assigned to the same cluster (i.e., the corresponding set of cannot-link constraints can no longer be satisfied). The dispersion then is the next higher distance than 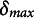 .

**Figure 1:**
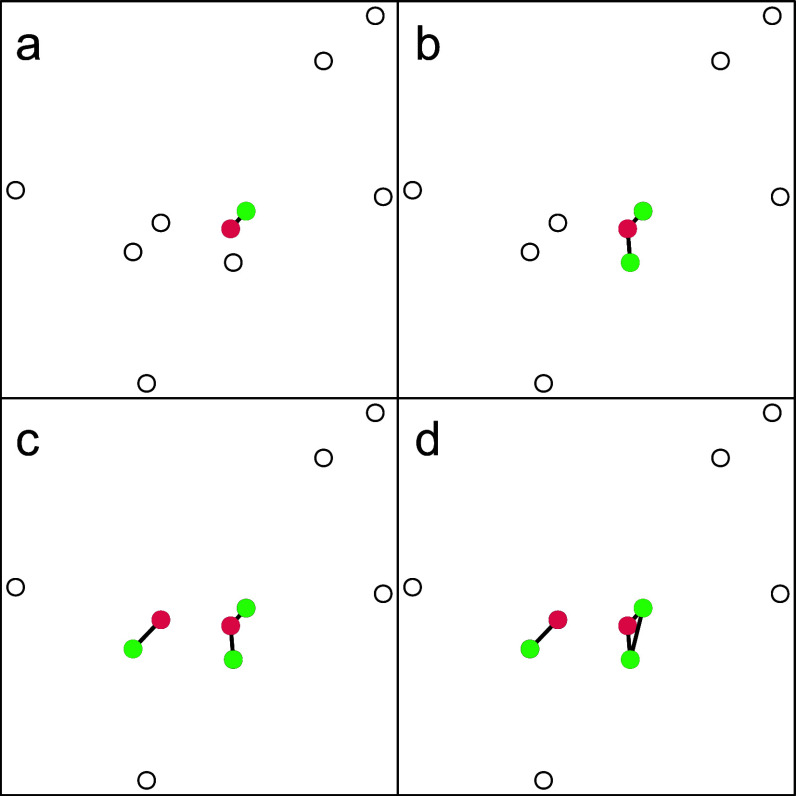
Illustrates the logic of the optimal search for the maximum dispersion for 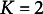 . *Note*: In Panel (a), the minimum distance between two points is identified and tested if the elements having it can be divided into two groups. It can be divided into two groups if the graph that consists of one edge can be 2-colored, i.e., is bipartite. After establishing bipartiteness, the next higher distance is identified and an edge is added to the graph between the elements that have it (Panel (b)). Again, the graph is tested for bipartiteness. The procedure of adding edges according to the order of increasing distances continues until the graph is no longer bipartite (Panel (d)). The last edge added in Panel (d) corresponds to the worst-case distance, i.e., the maximum dispersion. All data points that have not been colored are irrelevant for finding the maximum dispersion because all of their distances to other objects are larger than the dispersion. Hence, these remaining data points can be assigned to clusters arbitrarily (while respecting the cardinality constraints).

Our algorithm starts by setting 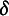 to the minimum of all dissimilarities, i.e., 
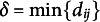
 . We then generate the cannot-link constraints corresponding to 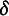 by creating a graph 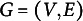 with *V* consisting of *N* vertices representing the *N* objects and initialize an empty set of edges *E*. We add an edge 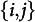 for each pair of objects *i* and *j* whose distance equals 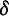 . We then test if the resulting graph is *K*-colorable (Davidson & Ravi, 2005). A graph is *K*-colorable if all adjacent vertices can be assigned different colors, while the maximum allowed number of colors is *K*. In our case, assigning different colors implies that objects whose dissimilarity is 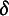 can indeed be assigned to *K* different groups. The *K*-coloring problem reduces to testing if a graph is bipartite for 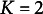 (Brucker, 1978). Because anticlustering applications apply fixed cardinality constraints on the number of objects per group, we employed an adaptation of the graph coloring model that restricts the number of times a color may be chosen (Méndez-Díaz et al., 2009, 2014). This way, we ensure that each solution to the *K*-coloring problem is valid regarding the cardinality constraints of the application. The full details of our graph coloring ILP model are found in the Appendix. If the graph is *K*-colorable while adhering to the cardinality restrictions, we increase 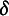 to the next higher value in our dissimilarity matrix, and again generate the corresponding constraints by adding all pairs of objects whose dissimilarity is 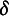 as edges to *G*, and test if the graph is still *K*-colorable. As long as the graph is *K*-colorable, we increase 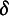 and each time test for *K*-colorability. As soon as the graph is no longer *K*-colorable, we set 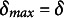 . Figure 1 illustrates the process of finding the maximum dispersion, highlighting that it can be found before all objects have been inspected; here, the maximum dispersion was found by inspecting only 5 of the 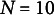 objects.

### 1.5 The exact bicriterion method

Based on the algorithm *OptDispF*, we developed an exact constraint method for the bicriterion model, which ensures that a partition with (optimal) maximum diversity cannot have lower dispersion than a pre-specified value. We refer to our method as *OptBicriterion*. Note that *OptBicriterion* is highly similar to the bicriterion partitioning method described by Brusco & Stahl (2005, pp. 82–84). The key differences are that they solved the converse clustering problem by minimizing the cluster diameter instead of maximizing the dispersion, and by minimizing within-group diversity instead of maximizing within-group diversity. Moreover, their approach did not incorporate cardinality constraints on the cluster sizes. Whereas cardinality constraints are oftentimes used in anticlustering methods, they are usually not applied in cluster analysis.

Our method *OptBicriterion* proceeds as follows. Using 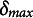 provided by *OptDispF*, we generate an adjusted distance matrix by setting dissimilarities for all objects *i* and *j* with 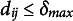 to 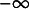 .^3^ This transformation of the dissimilarity matrix is already sufficient to ensure that any partition that is optimal with regard to the diversity cannot group objects *i* and *j* in the same cluster. If any such pair *i* and *j* were grouped in the same cluster, the diversity of the resulting partitioning would be 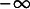 . However, at least one partition exists—the partition having maximum dispersion—where all object pairs with 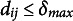 are in separate groups, and which has higher diversity than 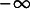 . This trick of adjusting a (dis)similarity matrix to induce cannot-link constraints is well-known in the context of optimal cluster editing (Böcker et al., 2011), which is the mathematical reversal of diversity anticlustering (Papenberg & Klau, 2021). By passing the adjusted dissimilarity matrix to the ILP method by Papenberg & Klau (2021), we optimally maximize the diversity under the constraint of preserving maximum dispersion. Generating the entire Pareto set is a straightforward extension of this logic: We successively decrease 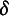 , adjust the dissimilarities 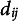 for each 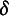 , and then compute the optimal diversity for the adjusted dissimilarity matrix. We store all partitions that are generated, remove potential duplicates, and drop all partitions that are not Pareto optimal, i.e., are dominated by a different partition.

### 1.6 Simulation 1: Feasibility of the exact bicriterion approach

To systematically investigate the computational feasibility of the exact bicriterion approach, we investigated the run time of *OptDispF* and *OptBicriterion*, which we implemented in anticlust (Version 0.8.10). We used the commercial software Gurobi (Version 12.0.1, Gurobi Optimization LLC, 2024) to solve the ILP models for equal-sized clusters. In the simulation, we restricted *OptBicriterion* to finding the maximum diversity on top of the globally optimal dispersion, instead of recovering the entire Pareto set. The simulation was implemented using the R programming language (version 4.4.2) on an Intel i7-10700 computer (4.800 GHz 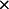 8) with 16 GB RAM, running Ubuntu 24.04.2 LTS x86_64.

We varied the sample size *N* and the number of *K*, whereas the number of variables was fixed at 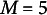 . All data were generated from a normal distribution ( 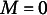 and *SD* = 1). Each combination of *K* and *N* was replicated 50 times to estimate the average run time. For each *K*, we increased *N* until one of two stopping criteria was met: either when a time limit was exceeded (we opted for 1,800 seconds following Schulz (2022)) or when the sample size exceeded 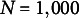 . Increasing *N* sequentially was implemented by the following rule: Starting with 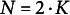 (e.g., 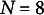 for 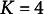 ), *N* was increased by *K* in each step for 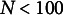 , by 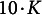 for 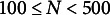 and by 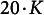 for 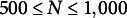 .

**Figure 2:**
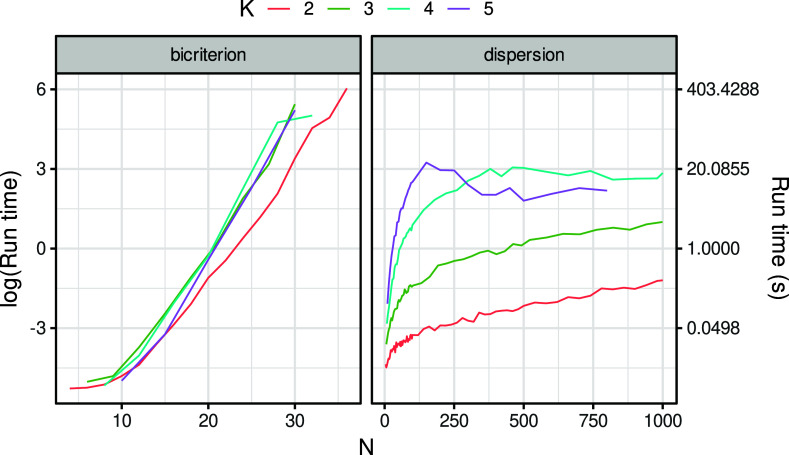
Average run time for the exact bicriterion approach (maximizing diversity while preserving optimal dispersion) and for optimally maximizing the dispersion alone. *Note*: Note that the *y*-axis is on a logarithmic scale and that the range of the *x*-axis differs between criteria.

Figure 2 shows that *OptDispF* was very quick in finding optimal solutions, especially for 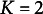 and 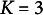 , where the optimal dispersion was usually found in less than 1 second. For 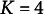 and 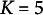 , we observe a stronger increase in run time with increasing *N*. Still, the time limit was never exceeded for 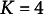 , and it only exceeded the time limit for 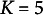 at 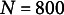 . The pattern of results looked very different for *OptBicriterion*, where the combinatorial explosion happened rather quickly. The maximum *N* that could be processed without exceeding the time limit was 36, 30, 32, and 30 for 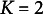 , 3, 4, and 5, respectively.

### 1.7 The hybrid bicriterion approach

Figure 2 shows that the fully exact bicriterion approach is only feasible for small to moderately sized data sets. However, the single criterion optimization of the dispersion could be applied to quite large data sets; arguably, most use cases in psychological research would be covered using the exact approach. To capitalize on the possibility of guaranteed optimal dispersion in the context of the bicriterion approach to anticlustering, we therefore developed the hybrid bicriterion approach. That is, we investigated how to utilize heuristic methods that maximize diversity while preserving the optimal dispersion returned by *OptDispF*. The BILS algorithm (Brusco et al., 2020) is a natural choice for this task because by keeping track of the Pareto set, it will never lose a partition that has a globally optimal value in dispersion when it has found one; such a partition can only be dominated by a partition that is also optimal with regard to the dispersion and better with regard to the diversity.

We generated the initializing partition for BILS as follows: We first apply *OptDispF*. As Figure 1 suggests, *OptDispF* does not necessarily have to assign each object to a group to fix the optimal dispersion. The remaining elements can be assigned randomly to groups without changing the dispersion. It is therefore possible to generate multiple different initializing partitions that are all globally optimal with regard to the dispersion. Hence, we can potentially initialize the MBPI with a different optimal partition for each restart. Whether it is preferable to just use one optimal partition for initialization—and use random partitions for the remaining runs—or if it is better to use multiple optimal partitions was investigated in Simulation 2 that compared different hybrid approaches.

We also developed a hybrid anticlustering approach based on a single criterion optimization of the diversity, which restricts the search space toward partitions that maintain optimal dispersion. To this end, we employed the LCW algorithm (Weitz & Lakshminarayanan, 1998) and initialized it with an optimal partition provided by *OptDispF*. Based on the optimal dispersion, we adjusted the dissimilarity matrix as we did for *OptBicriterion*, i.e., by replacing all 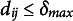 by 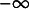 . During search, this manipulation of the dissimilarity matrix prevents LCW from accepting a pairwise exchange that would decrease the dispersion, because such an exchange would always be accompanied with a decrease in diversity. As compared to the hybrid BILS, the restricted LCW approach forbids some exploration of the search space all together, while BILS’s search is not restricted. BILS maintains the optimal dispersion of a partition by keeping track of the Pareto set, but otherwise moves through the space of partitions more freely.

### 1.8 Simulation 2: Comparison of hybrid bicriterion approaches

To find out which heuristic method best provides high diversity on top of an optimal dispersion, we conducted a simulation study that compared the different hybrid anticlustering algorithms. All anticlustering methods were implemented in the anticlust software (Version 0.8.10, Papenberg & Klau, 2021). Again, the Gurobi solver was used as the ILP backend of our exact algorithm *OptDispF*, and we used the same computer setup as in Simulation 1. The R packages dplyr (Version 1.1.4, Wickham et al., 2019), papaja (Version 0.1.2, Aust & Barth, 2018), ggplot2 (Version 3.5.1, Wickham, 2016), and tidyr (Version 1.3.1, Wickham, 2021) were used for data analysis. All code and data used in the simulation study can be retrieved from the accompanying OSF repository (https://osf.io/hsztn/).

In total, the simulation included four hybrid adaptations of the BILS and hybrid LCW. The four hybrid BILS adaptations resulted because (a) we varied if each run of the MBPI was initialized with a partition having optimal dispersion (*BILS-Hybrid-All*) or just the first (*BILS-Hybrid-1*) and (b) we used one BILS version that employed the ILS improvement procedure and one that only applied the initial local search phase of MBPI. The number of restarts was always set to 100; for the two methods that employed ILS, half of them conducted ILS. To generate initializing partitions for the hybrid BILS algorithms, we first applied *OptDispF* and randomly assigned the objects that were not fixed through the constraint of maximum dispersion (see Figure 1). For all BILS versions, we used the default values employed by Brusco et al. (2020) for the remaining parameters of the algorithm: For each restart, 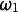 —which determines the relative weight of the diversity and dispersion criteria during search—was randomly selected from among ten values (0.000001, 0.00001, 0.0001, 0.001, 0.01, 0.1, 0.5, 0.99, 0.999, and 0.999999). The probability of swapping two objects during the ILS phase was uniformly sampled from the interval [5%, 10%].

To apply anticlustering using a varying number of groups 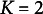 , 3, 4, and 5, 5,000 data sets were generated from a normal distribution for each *K*, totaling 20,000 data sets. The following parameters were selected randomly for each data set: (a) the sample size *N* varied between 20 and 120, imposing the restriction that *N* had to be divisible by *K* to create equal-sized groups via anticlustering; (b) the number of features varied between 2 and 5 with a population correlation of 0 for each pair of variables; and (c) the standard deviation of all features was set to 1, 2, or 3 (the mean was always 0). All six anticlustering methods were applied to each of the 20,000 data sets. For each BILS method, we retrieved the Pareto set and selected the partition having maximum—i.e., optimal—dispersion.

To quantify the performance of the competing methods, we used the following computations: First, we used average diversity as an indicator of the quality of the partitions across simulation runs. Second, we defined each solution as “good” or “not good” using a cutoff: Whenever the diversity of a partition was within 0.1% of the diversity of the highest diversity that was attained in this simulation run, it was defined as a “good” solution. Otherwise, it was defined as “not good.” The proportion of good solutions was used as a secondary indicator of the quality of the competing methods. Due to strong outliers in diversity, this additional measure provided crucial insights over and above what was portrayed in average diversity.

**Figure 3:**
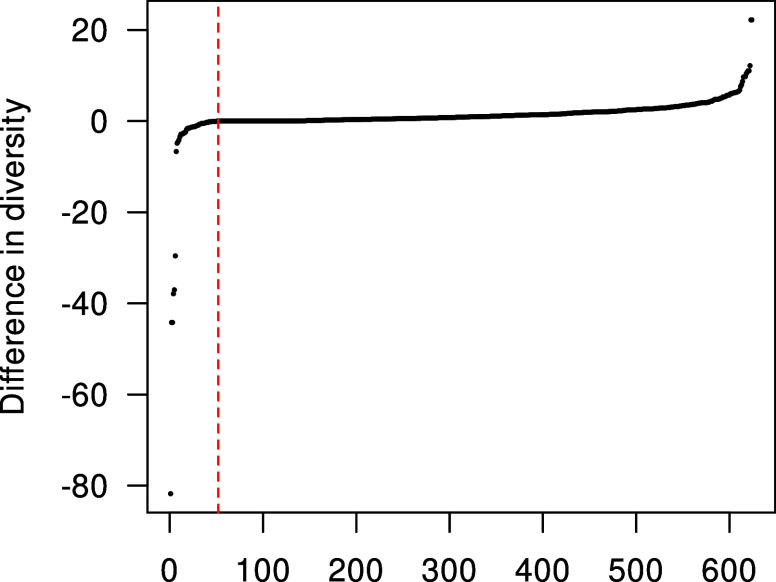
Sorted differences between the diversity returned by BILS-Hybrid-1-ILS and restricted LCW in data sets with maximum restriction. *Note*: The red vertical line highlights the turning point (difference of 0). While BILS-Hybrid-1-ILS more often had higher diversity than LCW, it sometimes returned far inferior partitions.

Table 1 displays the globally aggregated simulation results and the results in dependence of the number of groups *K*. Aggregated across all simulation runs, BILS-Hybrid-All with ILS performed best. It had the highest number of good solutions (98%) as well as the highest average diversity (4,351.18). For 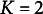 , all methods yielded comparable average diversity. For 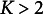 , the BILS-Hybrid-1 method obtained increasingly worse average diversity, while the constrained LCW algorithm obtained the highest average diversity. The pattern of results was, however, not unambiguously reflected through the proportion of good solutions; here, the LCW method tended to yield the worst results. Hence, while on average the LCW method found the most diverse partitions, it was less consistent in finding good solutions. In contrast, the BILS-Hybrid-1 methods oftentimes returned good solutions, even though their average performance was clearly subpar. During exploration of the results, it became evident that the BILS-Hybrid-1 methods sometimes tended to blunder and returned a far inferior partition. These outliers strongly decreased average diversity while the proportion of good solutions was on a similar level to the other methods (see Figure 3, which is discussed in more detail below). The BILS-Hybrid-All method that employed the ILS improvement phase offered the best performance across both quality measures: It produced the highest proportion of good solutions as well as high average diversity across simulation runs. Interestingly, using the ILS improvement led to considerably improved results as compared to only using the initial MBPI local search, especially for larger *K*.

**Table 1:** Results of the simulation study, grouped by *K*

		BILS-Hybrid-1		BILS-Hybrid-All		LCW
	*n*	MBPI	MBPI + ILS	MBPI	MBPI + ILS	
**Average diversity**						
*K* = 2	5,000	6,636.15	6,636.15	6,636.13	6,636.16	6,636.10
*K* = 3	5,000	4,681.36	4,681.38	4,681.48	4,681.50	4,681.53
*K* = 4	5,000	3,362.72	3,362.59	3,363.70	3,363.73	3,363.74
*K* = 5	5,000	2,719.66	2,720.15	2,723.25	2,723.33	2,723.26
Globally	20,000	4,349.98	4,350.07	4,351.14	4,351.18	4,351.16
**% Good solutions**						
*K* = 2	5,000	100.0	100.0	99.9	100.0	98.7
*K* = 3	5,000	99.6	99.5	98.3	99.6	97.0
*K* = 4	5,000	95.4	94.9	93.2	97.6	91.5
*K* = 5	5,000	87.2	87.3	87.6	94.7	84.8
Globally	20,000	95.6	95.4	94.7	98.0	93.0

*Note*: Shows the performance of the competing heuristics. A good solution was defined as a partition whose diversity was within 0.1% of the maximum diversity that was attained during a simulation run.

When exploring the results, we identified the factor that explained the complex pattern of results, i.e., the reason why the two quality measures did not always agree: the level of restriction that is induced by the constraint of maximum dispersion. To clarify, as suggested in Figure 1, it is oftentimes possible to randomly assign many objects to clusters while maintaining the same (optimal) dispersion. We used this feature to generate multiple different initializing partitions for the BILS-Hybrid-All and LCW methods. However, the number of objects that are fixed in their group to maintain the optimal dispersion depends on the data set. To quantify the degree of restriction that the constraint of maximum dispersion enforces in a particular data set, we computed the number of duplicate partitions that were generated as initialization of the BILS-Hybrid-All methods. If there was no duplicate, we labeled the corresponding data set with “no restriction”; if all 100 partitions were the same, we labeled the data set with “maximum restriction”; the remaining data sets were labeled with “some restriction.” As shown in Table 2, the most prevalent label was “no restriction,” followed by “some.” However, the restriction strongly depended on the number of groups *K*: For larger *K*, it was more likely that duplicate partitions were generated.

**Table 2:** Level of restriction in dependence of *K*

	Level of restriction			
	No	Some	Maximal	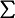
*K* = 2	4,572	425	3	5,000
*K* = 3	4,230	729	41	5,000
*K* = 4	3,551	1,286	163	5,000
*K* = 5	2,667	1,916	417	5,000
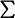	15,020	4,356	624	20,000

Table 3 displays the performance of the hybrid algorithms by level of restriction. In data sets with no restriction, the restricted LCW method was the best method: It obtained the highest diversity and was tied with regard to the number of good solutions. However, the performance of LCW strongly decreased when data sets became more restricted. When the level of restriction increased, the BILS methods performed better. Interestingly, the BILS-Hybrid-1 methods had the highest proportion of good solutions when there was maximum restriction, but still yielded the worst average diversity. Figure 3 displays the reason for this discrepancy, by plotting the difference in diversity between LCW and BILS-Hybrid-1 with ILS in data sets with maximum restriction. BILS-Hybrid-1 had higher diversity than LCW in the vast majority of comparisons (79.65%), but had slightly lower average diversity (536.33 vs. 535.36). The discrepancy is explained by the strong outliers shown in Figure 3. Sometimes, BILS-Hybrid-1 provided far inferior results. The application of BILS-Hybrid-1 is therefore not recommended.

**Table 3:** Results of the simulation study, grouped by level of restriction

		BILS-Hybrid-1		BILS-Hybrid-All		LCW
	*n*	MBPI	MBPI + ILS	MBPI	MBPI + ILS	
**Average diversity**						
No restriction	15,020	5,426.63	5,426.72	5,427.77	5,427.74	5,427.84
Some restriction	4,356	1,183.88	1,183.96	1,185.38	1,185.50	1,185.25
Maximal restriction	624	536.22	536.33	535.71	536.71	535.36
**% Good solutions**						
No restriction	15,020	97.8	97.8	99.8	99.7	99.8
Some restriction	4,356	88.7	88.2	85.6	94.2	78.8
Maximal restriction	624	88.3	87.2	37.5	83.0	28.7

*Note*: Shows the performance of the competing heuristics. A good solution was defined as a partition whose diversity was within 0.1% of the maximum diversity that was returned during a simulation run.

### 1.9 Example application

In an example application, we illustrate the usage of the hybrid anticlustering approaches. The full code and data to reproduce these examples with the anticlust R package are available from the accompanying OSF repository (https://osf.io/hsztn/). We employed a set of word stimuli used by Schaper et al. (2019b; 2019a), consisting of 96 words that are typically associated with bathrooms (*n* = 48) or with kitchens (*n* = 48). For their experiment on source memory, each stimulus set was divided into three equal-sized lists, respectively, with equal frequency of each room type. Lists either served as stimuli during the learning phase or as distractors in a test phase, and lists should be comparable on typicality ratings, word frequency, and number of syllables. In the following, we outline how to obtain balance with regard to the covariates between lists; using our hybrid methods, we illustrate how to simultaneously maximize pairwise orthographic dissimilarity within lists.

We used two dissimilarity matrices to compute the dispersion and diversity, respectively. The first dissimilarity matrix 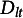 was used to maximize dispersion; it was computed as the normalized Levenshtein distance, representing pairwise orthographic dissimilarity of the word stimuli (Yujian & Bo, 2007). The second dissimilarity matrix 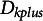 was used to maximize the diversity; it was computed as the pairwise squared Euclidean distance based on typicality ratings, number of syllables, and word frequency, and room type (binary coded). Before computing the squared Euclidean distances, the numeric variables (typicality ratings, number of syllables, word frequency) were extended via *k*-plus variables (Papenberg, 2024). Thus, via (8), maximizing the diversity on this dissimilarity matrix performed *k*-plus anticlustering, which maximizes similarity with regard to means and standard deviations of the input features. In the case of the binary coded room type, the mean is equivalent to the proportion of room types.

We divided the stimulus set of 96 words into three groups of 32 words, respectively. First, we applied *OptDispF* on the normalized Levenshtein distances. The optimal dispersion was 0.47, which, for example, corresponds to the orthographic dissimilarity between the German words “Nagelfeile” (nail file) and “Badmuelleimer” (bathroom trash bin). Thus, for a stimulus assignment with optimal maximum dispersion, the worst-case within-list dissimilarity corresponds to the dissimilarity between these words. On the basis of the output of *OptDispF*, we generated 10,000 equal-sized partitions having optimal dispersion. The 10,000 partitions contained no duplicates; hence, the data set constitutes an unrestricted hybrid problem. We applied three hybrid methods (restricted LCW, BILS-Hybrid-1, and BILS-Hybrid-All) and vanilla BILS with 10,000 restarts, respectively. For each BILS method, we used 5,000 restarts of MBPI and 5,000 restarts of ILS. Though Simulation 2 indicated that the ILS phase does not improve results for unrestricted hybrid problems, we generally recommend using it because the structure of the data is usually not known in advance. Moreover, ILS always has the potential to improve upon results from MBPI. Hence, instead of dropping ILS entirely, it would be more reasonable to increase the total number of BILS iterations, so that a sufficient number of MBPI restarts are conducted before the ILS improvement phase proceeds.

**Figure 4:**
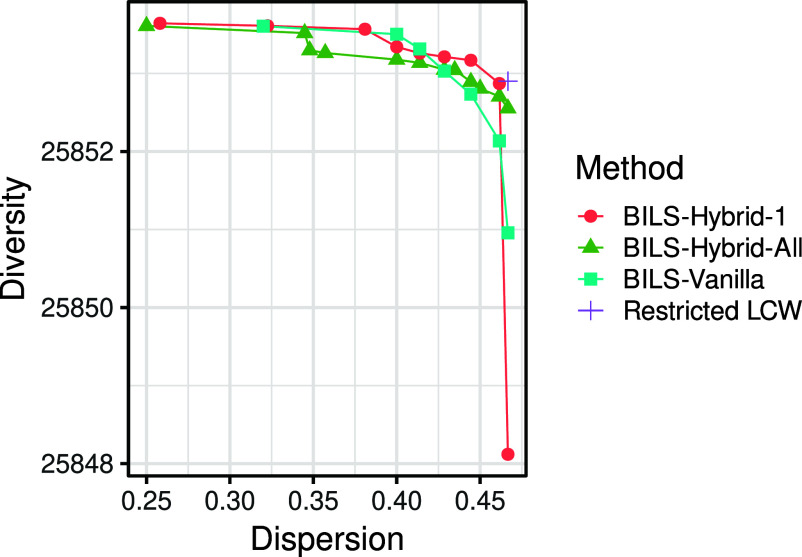
Illustrates the objective values of the hybrid anticlustering methods applied on the data set by Schaper et al. (2019a; 2019b).

Figure 4 shows the criterion values returned by the four methods. For each BILS method, the entire Pareto set returned is depicted as a curve, which is a common depiction of Pareto sets and was also used by Brusco et al. (2020) to display the results of BILS. As LCW returns a single partition, its results are depicted using a single data point. Note that vanilla BILS found a partition having optimal dispersion, which was not guaranteed by this method. The diversities of the four partitions with optimal dispersion were 25,852.9 (Restricted LCW), 25,852.55 (BILS-Hybrid-All), 25,850.96 (BILS-Vanilla), and 25,848.12 (BILS-Hybrid-1). Consistent with the simulation results, BILS-Hybrid-All and restricted LCW yield the highest diversity on top of optimal dispersion. BILS-Hybrid-1 and vanilla BILS provided considerably worse diversity for the partition that had optimal dispersion.

Table 4 illustrates the descriptive statistics for the partition that had overall highest diversity (returned by BILS-Hybrid-1) and the partition that provided highest diversity on top of optimal dispersion (returned by Restricted LCW). Arguably, between-group similarity is the same for both partitions. However, these partitions differ with regard to dispersion in practically meaningful terms: The partition with overall highest diversity had a dispersion of 0.26, which, for example, corresponds to the orthographic similarity of the German words Gesichtswasser (face lotion) and Gesichtspuder (face powder). These words are orthographically and semantically highly similar and could easily be confused in a memory experiment, possibly confounding the measurement of memory performance (Sekuler & Kahana, 2007). The worst case orthographic dissimilarity in partitions with maximum dispersion (nail file vs. bathroom trash bin) does not suffer from this potential problem.

**Table 4:** Between-group similarity for partitions having optimal dispersion (returned by Restricted LCW) and maximum diversity (returned by BILS-Hybrid-1) using the data set provided by Schaper et al. (2019a; 2019b)

	Typicality	Atypicality	Syllables	Frequency	Bathroom	Kitchen
Maximum diversity						
List 1	4.49 (0.25)	1.10 (0.07)	3.41 (0.95)	18.31 (2.40)	16	16
List 2	4.49 (0.25)	1.10 (0.07)	3.41 (0.95)	18.31 (2.42)	16	16
List 3	4.49 (0.25)	1.10 (0.07)	3.44 (0.91)	18.31 (2.42)	16	16
Optimal dispersion						
List 1	4.49 (0.25)	1.10 (0.07)	3.41 (0.95)	18.31 (2.42)	16	16
List 2	4.49 (0.25)	1.10 (0.07)	3.41 (0.95)	18.31 (2.42)	16	16
List 3	4.50 (0.25)	1.10 (0.07)	3.44 (0.91)	18.31 (2.40)	16	16

*Note*: Means and standard deviations (in parentheses) are given for the numeric variables Typicality, Atypicality, Syllables, and Frequency. Counts are given for the categorical variable room type (bathroom and kitchen).

We would like to highlight that while BILS-Hybrid-All performed best in Simulation 2 with regard to maximizing the diversity of the dispersion-maximizing partition, Figure 4 suggests that it does not improve the overall Pareto optimality of the BILS algorithm. BILS-Hybrid-1 and the original BILS overall discovered partitions with higher diversity. BILS-Hybrid-All instead seemed to nudge the search algorithm to improve upon the partition having optimal dispersion, potentially at the cost of finding overall higher diversity. While we argue that in the current case it is useful to select the dispersion-maximizing partition—as it does not really come at a cost of between-group similarity—there may be data sets where the optimal dispersion does induce a greater cost for between-group similarity. In these cases, BILS-Hybrid-All may not be the best choice.

### 1.10 Discussion

Anticlustering is a partitioning method that leads to similarity between groups and heterogeneity within groups. Many applications in psychology focus on obtaining groups that are similar with regard to relevant covariates, e.g., when assigning stimuli to sets in within-subjects experiments (Lintz et al., 2021; Schaper et al., 2023). Brusco et al. (2020) noted that a sole focus on group-level characteristics overlooks that dissimilarity on the item level can usually be achieved without losing out on between-group similarity. They presented an anticlustering algorithm that simultaneously optimizes for both criteria (BILS). In an example application using a data set by Schaper et al., 2019a (2019a, 2019b; see also Papenberg & Klau, 2021), we illustrated a context where their bicriterion approach is practically useful: In recognition memory experiments, it might be desirable to maximize within-list orthographic dissimilarity to avoid unwarranted confusion among stimuli, thus ensuring valid measures of memory performance (Sekuler & Kahana, 2007). At the same time, it is desirable to equate variables that affect overall memory performance (Lintz et al., 2021). Using our novel hybrid anticlustering method, we achieved high balance on covariates while simultaneously achieving low confusability of stimuli. Just striving for similarity between lists did not meaningfully improve group-level balance, but led to considerably increased confusability.

In this article, we extended the bicriterion approach for anticlustering by Brusco et al. (2020) in several ways. First, our implementation of their BILS algorithm allows that the diversity and dispersion can be computed on the basis of two different dissimilarity matrices. Thus, different kinds of information can be employed for both criteria, which was beneficial in our example application. As another contribution, we recognized the close connection of the *k*-means and diversity criteria for anticlustering (see (6)–(8)). Consequently, we ensured that our implementation of the BILS algorithm can also optimize the *k*-means and *k*-plus anticlustering criteria as measures of between-group similarity, instead of only the diversity (Papenberg, 2024).

As our most important contribution, we developed a model that optimally solves the bicriterion anticlustering approach, consisting of the algorithms *OptDispF* and *OptBicriterion*. *OptDispF* optimally partitions data sets with the objective of obtaining maximum dispersion. It can be applied to large data sets despite the NP-hardness of the maximum dispersion problem (see Figure 2). Note that the maximum dispersion problem involving only two groups ( 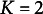 ) is not NP-hard when no cardinality constraints are imposed. This result has been shown repeatedly for the converse clustering problem (Brucker, 1978; Rao, 1971), and it also follows directly from the logic of 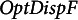 , which would run in polynomial time if it were sufficient to repeatedly test if a graph is bipartite without ensuring that the cardinality constraints are met. For 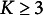 , constrained and unconstrained versions of the maximum dispersion problem are NP-hard (e.g., Fernández et al., 2013). To the best of our knowledge, it remains an open question if the maximum dispersion problem for 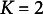 is NP-hard when cardinality constraints are imposed.

Based on the results provided by 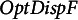 , *OptBicriterion* applies a constraint method that maximizes diversity while ensuring that the maximum level of dispersion is not exceeded (see also Brusco & Stahl, 2005). Because the fully exact approach *OptBicriterion* was shown to only scale to rather small data sets, we also developed hybrid approaches (BILS-Hybrid-All, BILS-Hybrid-1, and Restricted LCW). The hybrid approaches utilize *OptDispF*, but employ heuristic algorithms to maximize the diversity on top of an optimal dispersion.

We developed several hybrid extensions of the original BILS method. In the most simplest adaptation, we initialized BILS with a single partition that is optimal with regard to the dispersion. Because the BILS keeps track of the Pareto set, it will also output a partition with optimum dispersion, which is however likely improved over the initial partition with regard to diversity. Our simulation study showed that it is actually preferable to initialize each restart of the BILS with a different initial partition that is optimal with regard to the dispersion. That is, BILS-Hybrid-All outperformed BILS-Hybrid-1. As another hybrid approach, we developed an adaptation of the LCW algorithm, which is a single criterion optimization algorithm. We initialize the LCW search with one or multiple optimal partitions provided by *OptDispF*; by adjusting pairwise distance between objects that must not be assigned to the same cluster, the search process is then restricted toward partitions that maintain optimal dispersion. The restricted LCW algorithm also provided strong results in the simulation, but showed decreased performance when maintaining maximum dispersion restricted the search process too severely. In this case, BILS proved to be more flexible in its search for improved partitions.

We provide several recommendations for practical researchers who wish to apply our bicriterion anticlustering algorithms. First, based on Simulation Study 2, we recommend addressing bicriterion anticlustering problems using the BILS-Hybrid-All algorithm with both phases (MBPI + ILS), preferably using many restarts. This method provided the most robust results and is expected to be a reasonable choice in most settings. However, note that for anticlustering, it is usually possible to just try out different methods and select the best result post-hoc. It is entirely reasonable to try out different adaptations of BILS (as well as LCW) and to investigate the resulting partitionings according to the descriptive statistics of all variables, the dispersion, or even regarding any secondary criteria that were not part of the optimization process. The code underlying our practical example is available from the accompanying OSF repository and can be adapted by users for their purposes. We would also like to remind readers that all BILS methods return a Pareto set of partitions and not a single partition (see Figure 4). If the dispersion-maximizing partition is satisfactory with regard to between-group similarity, researchers may just choose this partition—as we did in our practical example (see Table 4). However, sometimes there is a non-trivial tradeoff between the dispersion and between-group similarity. In this case, researchers should inspect the resulting partitions, plot the Pareto set, and thoughtfully decide which partition should be chosen. Brusco et al. (2020) also provided guidelines on how to select a partition from the Pareto set returned by BILS.

In the end, we note that our exact algorithm *OptDispF* provides a general method to include cannot-link constraints with anticlustering (Davidson & Ravi, 2005). That is, maximizing the dispersion is only one possibility to enforce such constraints: Based on a threshold on pairwise dissimilarity, pairs of objects are forbidden from being assigned to the same cluster. However, using the graph coloring model that is the critical step in *OptDispF* (see the Appendix), inducing any kinds of user-defined cannot-link constraints is straightforward. We enable these constraints with a user-friendly interface in our anticlust package: As of version 0.8.6, the main function anticlustering() has an argument cannot_link that users can provide with pairs of objects that should not be assigned to the same cluster. If the set of constraints cannot be satisfied, the function will output an error.

#### 1.10.1 Conclusion

In this article, we provided several extensions of the innovative bicriterion approach for anticlustering by Brusco et al. (2020), enhancing its applicability and ensuring optimal results with regard to one or both criteria. All methods presented here are freely available via the open-source R package anticlust (https://CRAN.R-project.org/package=anticlust). The package website (https://github.com/m-Py/anticlust) provides additional community support and documentation.

## Data Availability

All code and data to reproduce all analyses presented in this article are available from the Open Science Repository via https://osf.io/hsztn/.

## Footnotes

1 In the context of optimization algorithms, it is usually more efficient to minimize  instead of maximizing  because its computation requires investigating only the cluster centroids and not the individual data points.

2 A single-object cluster relocation cannot be applied to maximize the diversity  , because the diversity is maximized by putting all objects into the same cluster. Single-object cluster relocation works for the *k*-means criterion  because it normalizes the within-cluster sums by the cluster sizes.

3 In practice, a sufficiently large negative value suffices. We used the negative sum of all dissimilarities minus 1.

## Appendix

We used an ILP model to test if a graph is *K*-colorable as the critical step of our exact algorithm for maximum dispersion (  ). It is an adaptation of the equitable graph coloring ILP by Méndez-Díaz et al. (2009, 2014). Our model has binary assignment variables  for each color  , with  if a color is used and  otherwise. Next, for each node  and each color  , we introduce assignment variables  with  if a vertex *v* is given color *k* and  otherwise. The complete ILP model is defined as

(A1)

(A2)

(A3)

(A4)

(A5)

(A6)

The model minimizes the colors used (1), ensures that each vertex is assigned exactly one color (2), that neighboring vertices are assigned different colors (3), and that the cardinality constraints are respected (4). Constraints (5) and (6) ensure that all decision variables are binary. The minimization (1) is standard for graph coloring models, but in principle, it is not needed here—it is sufficient to know whether the constraints can be fulfilled, as the optimization of the objective of interest (maximizing the dispersion) takes place outside of the graph coloring model.

There are three differences of our model to the model by Méndez-Díaz et al (2009; 2014). First, our model only requires upper bounds on the number of times a color is used, whereas their model specifies upper bounds and lower bounds. Second, we only allow *K* different colors, whereas their model uses up to *N* colors. When minimizing the objective function (1) while allowing *N* as the maximum number of colors, the so-called chromatic number is obtained, i.e., the minimum number of colors needed to color the graph. However, we are not interested in the chromatic number, but only whether the graph can be colored using *K* colors. As a third and last difference, we dropped an additional set of constraints used by Méndez-Díaz et al. for symmetry breaking. Symmetry breaking is a technique to prevent the occurrence of equivalent solutions, which can be crucial in many kinds of graph coloring problems. Specifically, in graph coloring, colors are interchangeable. In the sense of the ILP model, however, exchanging two colors with one another exhibits different solutions (i.e., constellations of the decision variables), even if they constitute the same grouping of nodes. To prevent the solver from testing symmetric solutions, which may increase running time, an additional set of constraints can be employed:

In this case, color  can only be used if color *j* has also been used.

In the early development of our method, we implemented the symmetry breaking constraints, but they tended to increase rather than decrease run time. The cost of including the additional constraints seemed to outweigh the benefits of symmetry breaking. We presume this is because we only allowed *K* colors and *K* was usually small for the practical problems we were interested in. The effect of symmetry breaking is much stronger if *N* colors can be used, because the number of symmetric solutions increases factorially with the number of possible colors.
